# Chronic OVA allergen challenged Siglec-F deficient mice have increased mucus, remodeling, and epithelial Siglec-F ligands which are up-regulated by IL-4 and IL-13

**DOI:** 10.1186/1465-9921-11-154

**Published:** 2010-11-01

**Authors:** Jae Youn Cho, Dae Jae Song, Alexa Pham, Peter Rosenthal, Marina Miller, Shanna Dayan, Taylor A Doherty, Ajit Varki, David H Broide

**Affiliations:** 1 Department of Medicine, University of California San Diego, San Diego, California, USA

## Abstract

**Background:**

In this study we examined the role of Siglec-F, a receptor highly expressed on eosinophils, in contributing to mucus expression, airway remodeling, and Siglec-F ligand expression utilizing Siglec-F deficient mice exposed to chronic allergen challenge.

**Methods:**

Wild type (WT) and Siglec-F deficient mice were sensitized and challenged chronically with OVA for one month. Levels of airway inflammation (eosinophils), Siglec-F ligand expresion and remodeling (mucus, fibrosis, smooth muscle thickness, extracellular matrix protein deposition) were assessed in lung sections by image analysis and immunohistology. Airway hyperreactivity to methacholine was assessed in intubated and ventilated mice.

**Results:**

Siglec-F deficient mice challenged with OVA for one month had significantly increased numbers of BAL and peribronchial eosinophils compared to WT mice which was associated with a significant increase in mucus expression as assessed by the number of periodic acid Schiff positive airway epithelial cells. In addition, OVA challenged Siglec-F deficient mice had significantly increased levels of peribronchial fibrosis (total lung collagen, area of peribronchial trichrome staining), as well as increased numbers of peribronchial TGF-β1+ cells, and increased levels of expression of the extracellular matrix protein fibronectin compared to OVA challenged WT mice. Lung sections immunostained with a Siglec-Fc to detect Siglec-F ligand expression demonstrated higher levels of expression of the Siglec-F ligand in the peribronchial region in OVA challenged Siglec-F deficient mice compared to WT mice. WT and Siglec-F deficient mice challenged intranasally with IL-4 or IL-13 had significantly increased levels of airway epithelial Siglec-F ligand expression, whereas this was not observed in WT or Siglec-F deficient mice challenged with TNF-α. There was a significant increase in the thickness of the peribronchial smooth muscle layer in OVA challenged Siglec-F deficient mice, but this was not associated with significant increased airway hyperreactivity compared to WT mice.

**Conclusions:**

Overall, this study demonstrates an important role for Siglec-F in modulating levels of chronic eosinophilic airway inflammation, peribronchial fibrosis, thickness of the smooth muscle layer, mucus expression, fibronectin, and levels of peribronchial Siglec-F ligands suggesting that Siglec-F may normally function to limit levels of chronic eosinophilic inflammation and remodeling. In addition, IL-4 and IL-13 are important regulators of Siglec-F ligand expression by airway epithelium.

## Background

Siglec-F **(Si**alic acid-binding **Ig**-superfamily **lec**tin-F) belongs to the CD33-related Siglec (CD33rSiglec) family which are a subclass of Siglecs defined by their mutual sequence similarity and clustered gene localization (chromosome 7 in mouse; chromosome 19q in humans) [1]. Eosinophils express a restricted profile of Siglecs [2-5]. Of the eight mouse Siglecs and fourteen human Siglecs that have been identified, eosinophils are reported to highly express significant levels of Siglec-F in mice [2-5] and its functionally convergent ortholog Siglec-8 in human eosinophils [6-8]. Most of the CD33rSiglecs are expressed on cells involved in innate immunity, such as monocytes, granulocytes, macrophages and natural killer cells [1]. Siglec-F is a transmembrane receptor comprising a ligand binding V-set domain, three C-2 domains, a transmembrane domain, and a cytoplasmic ITIM motif (immunoreceptor tyrosine-based inhibitory motif), which is known to be involved in inhibitory signaling pathways in the immune system [9,10]. Support for inhibitory signaling by the cytoplasmic domain of CD33rSiglecs have come from studies which have demonstrated that antibody cross-linking of several CD33rSiglecs results in inhibition of cellular-activation signals, arrest of proliferation, or induction of apoptosis [11-13].

Siglec-F is highly expressed on mouse eosinophils [5] and levels of Siglec-F are up-regulated on peripheral blood eosinophils following acute OVA challenge in wild type (WT) mice [5]. We have generated Siglec-F deficient mice and demonstrated that these mice have similar baseline levels of peripheral blood eosinophils as do WT mice [5]. However, following acute OVA challenge Siglec-F deficient mice have significantly increased numbers of eosinophils in the bone marrow, blood, and lung compared to WT mice [5]. These studies in Siglec-F deficient mice suggest that Siglec-F plays an inhibitory role in acute eosinophilic inflammation. Studies with an anti-Siglec-F Ab have demonstrated that it reduces levels of eosinophilic inflammation and induces eosinophil apoptosis when administered in mouse models of gastro-intestinal eosinophilic inflammation [14], lung eosinophilic inflammation [15], or a mouse model of the hypereosinophilic syndrome [16]. Although studies have examined the role of Siglec-F utilizing Siglec-F deficient mice in acute antigen challenge models of asthma [5], studies have not utilized Siglec-F deficient mice to examine whether Siglec-F plays a role in chronic antigen induced airway remodeling which is the focus of this study. As eosinophils may contribute to airway remodeling [7,17], we examined whether Siglec-F deficient mice would have increased levels of airway remodeling, and deposition of extracellular matrix proteins in the airway in vivo. In addition, as in previous studies we have demonstrated that WT mice challenged with allergen have increased levels of expression of Siglec-F ligands in the airway epithelium and peribronchial cells [3,5], we examined whether the absence of Siglec-F receptors in Siglec-F deficient mice would modulate levels of Siglec-F ligands expressed in the airway of Siglec-F deficient compared to WT mice.

## Methods

### Mouse Model of Chronic OVA-induced Eosinophilic Inflammation and Airway Remodeling

The mouse model of OVA induced airway remodeling has previously been described [7,18]. In brief, eight-to ten-wk-old Siglec-F deficient mice (n = 16/group)[5] and WT mice (n = 16/group) on a background of C57/Black were immunized sc on days 0, 7, 14, and 21 with 25 µg of OVA (grade V, Sigma) adsorbed to 1 mg of alum (Aldrich) in 200 µl of normal saline. Intranasal OVA challenges (20 µg/50 µl in PBS) were administered on days 27, 29, and 31 under isoflurane (Vedco, St. Joseph, MO) anesthesia. Intranasal OVA challenges were then repeated twice a week for 4 weeks. Age-and sex-matched control mice were sensitized but not challenged with OVA during the study. Mice were sacrificed 24 h after the final OVA challenge and bronchoalveolar lavage (BAL) fluid was collected by lavaging the lung with 1 mL PBS via a tracheal catheter [5]. Lungs from the different experimental groups were processed as a batch for either histological staining or immunostaining under identical conditions. Stained and immunostained slides were all quantified under identical light microscope conditions, including magnification (x20), gain, camera position, and background illumination. All animal experimental protocols were approved by the University of California, San Diego Animal Subjects Committee.

### Blood, Bone marrow, BAL, and peribronchial eosinophils

Peripheral blood was collected from mice by cardiac puncture into EDTA-containing tubes as previously described [5]. Erythrocytes were lysed using a 1:10 solution of 100 mM potassium carbonate-1.5 M ammonium chloride [5]. The remaining cells were re-suspended in 1 mL PBS. Bone marrow cells were flushed from femurs with 1 mL PBS, centrifuged, and re-suspended in 1 mL PBS as previously described [5]. BAL was collected by lavaging the lung with 1 mL PBS via a tracheal catheter as previously described [5]. BAL was centrifuged, the supernatant was collected and frozen at -80°C, and cells were re-suspended in 1 mL PBS. Total leukocytes were counted using a hemocytometer. To perform differential cell counts, 200 µL re-suspended BAL cells, peripheral-blood leukocytes, or 20 µL bone marrow cell suspensions was cytospun onto microscope slides and air-dried. Slides were stained with Wright-Giemsa and differential cell counts were performed under a light microscope [5].

Lung sections were processed for major basic protein (MBP) immunohistochemistry as previously described [5], using an anti-mouse MBP Ab (kindly provided by James Lee PhD, Mayo Clinic, Scottsdale, Arizona). The number of individual cells staining positive for MBP in the peribronchial space were counted using a light microscope. Results are expressed as the number of peribronchial cells staining positive for MBP per bronchiole with 150-200 µm of internal diameter. At least ten bronchioles were counted in each slide.

### Airway mucus expression

To quantitate the level of mucus expression in the airway, the number of periodic acid Schiff (PAS)-positive and PAS-negative epithelial cells in individual bronchioles were counted as previously described in this laboratory [7]. At least ten bronchioles were counted in each slide. Results are expressed as the percentage of PAS-positive cells per bronchiole, which is calculated from the number of PAS-positive epithelial cells per bronchus divided by the total number of epithelial cells of each bronchiole.

### Peribronchial trichrome staining

Lungs in the different groups of mice were equivalently inflated with an intratracheal injection of the same volume of 4% paraformaldehyde solution (Sigma Chemicals, St. Louis, MO) to preserve the pulmonary architecture. The area of peribronchial trichrome staining in paraffin-embedded lungs was outlined and quantified under a light microscope (Leica DMLS, Leica Microsystems) attached to an image analysis system (Image-Pro plus, Media Cybernetics) as previously described [7]. Results are expressed as the area of trichrome staining per µm length of basement membrane of bronchioles 150-200 µm of internal diameter.

### Lung collagen assay

The amount of lung collagen was measured as previously described in this laboratory (8) with a collagen assay kit that uses a dye reagent that selectively binds to the [Gly-X-Y]n tripeptide sequence of mammalian collagens (Biocolor, Newtonabbey, UK). In all experiments, a collagen standard was used to calibrate the assay.

### Peribronchial TGF-β1 + cells

The number of peribronchial TGFβ-1+ cells were quantitated by immunohistochemistry using an anti-TGF-β1 Ab as previously described in this laboratory [7].

### Lung cytokines and chemokines

Levels of lung cytokines (IL-5, IL-13) and lung chemokines (eotaxin-1, RANTES) were quantitated by ELISA (R&D Systems, Minneapolis, MN) in lung tissue as previously described [18]. In brief, lung tissue was homogenized in lysis buffer, and lung supernatants (obtained by centrifugation 10,000 *g *for 20 min) were passaged through an 0.8-µm pore size filter and frozen at-80°C in polypropylene tubes until used in assays [18]. The IL-5, IL-13, and RANTES ELISA assays each had a sensitivity of 31 pg/ml, and the eotaxin-1 assay a sensitivity of 16 pg/ml.

### Lung LTC4 levels

As leukotriene C4 (LTC4) is a product of eosinophils [19], and LTC4 is associated with airway remodeling [20], we determined levels of LTC4 in lung tissue using a using a LTC4 ELISA (Neogen, Lexington, KY). The sensitivity of the LTC4 assay was 0.04 ng/ml.

### Detection of extracellular matrix protein fibronectin

Lung sections were processed for fibronectin immunohistochemistry using a rabbit anti-mouse fibronectin Ab (Abcam, Cambridge, MA). The area of peribronchial fibronectin staining was outlined and quantified under a light microscope (Leica DMLS) attached to an image analysis system (Image-Pro plus) as described for trichrome staining. Results are expressed as the area of fibronectin staining per µm length of basement membrane of bronchioles 150-200 µm of internal diameter.

### Siglec-F ligand expression

Cryostat sections of lung tissues were incubated with a recombinant Siglec-F-Fc (or as a negative control R114A Siglec-F-Fc) and detected using the immunoperoxidase method as previously described [5]. R114A Siglec-F-Fc is a specific negative control comprising the same Siglec-F-Fc with a single amino acid mutation (i.e. an Arg-Ala mutation that eliminates sialic acid recognition)[5]. The Siglec-F-Fc comprises the extracellular domains of Siglec-F involved in sialic acid recognition fused with the Fc portion of human IgG. This probe can be used to detect specific ligands on cells and in tissue sections [5]. In previous studies in WT mice we have demonstrated that following OVA challenge Siglec-F ligands are expressed in airway epithelium and peribronchial cells (eosinophils, macrophages)[5]. Following lung immunostaining, the area of epithelial Siglec-F-Fc immunostaining was outlined and quantified using a light microscope (Leica DMLS) attached to an image-analysis system (Image-Pro Plus). Results are expressed as the area of epithelial immunostaining per µm length of epithelial basement membrane of bronchioles with 150-250 µm internal diameter. At least ten bronchioles were counted in each slide. In addition to quantitating the levels of Siglec-F ligand expression by airway epithelial cells, we also quantitated the number of peribronchial cells expressing Siglec-F ligands. Results are expressed as the number of peribronchial cells staining positive for Siglec-F-Fc per bronchiole with 150-200 µm of internal diameter.

### Effect of cytokines on Siglec-F Ligand expression in WT mice

Eight week old WT C57BL/6 mice or Siglec-F deficient mice (n = 3/group) were challenged intranasally with 0.6 µg of each individual cytokine (IL-4, IL-13, TNF-α, or PBS diluent control) (R&D Systems, Minneapolis, MN) under isofluorane anesthesia using a modification of a previously published protocol [21]. The dose of cytokine administered intranasally was chosen based on dosages used in studies in a previously published protocol [21]. Twenty four hours after each individual cytokine or diluent challenge, the mice were sacrificed. BAL was obtained for determination of eosinophil and neutrophil cell counts, and the lungs were processed for immunohistology to detect Siglec-F ligand expression in airway epithelium and peribronchial MBP+ eosinophils as described above.

### Peribronchial Smooth Muscle Layer Thickness

Lung sections were also immunostained with an anti-α-smooth muscle actin primary antibody (Sigma-Aldrich) to detect peribronchial smooth muscle cells as previously described in this laboratory [22]. Species-and isotype-matched Abs were used as controls in place of the primary Ab. The area of peribronchial α-smooth muscle actin staining in paraffin-embedded lungs was outlined and quantified under a light microscope (Leica DMLS) attached to an image analysis system (Image-Pro plus) as previously described [7]. Results are expressed as the area of peribronchial α-smooth muscle actin staining per µm length of basement membrane of bronchioles 150-200 µm of internal diameter.

### Airway hyperreactivity to Mch

Airway responsiveness to methacholine (MCh) was assessed 24 h after the final OVA challenge in intubated and ventilated mice (flexiVent ventilator; Scireq, Montreal, PQ, Canada) anesthetized with ketamine (100 mg/kg) and xylazine (10 mg/kg) intraperitoneally as previously described [23]. The dynamic airway resistance (Raw) was determined using Scireq software in mice exposed to nebulized PBS and MCh (3, 24, 48 mg/ml). The following ventilator settings were used: tidal volume (10 ml/kg), frequency (150/min), positive end-expiratory pressure (3 cmH_2_O).

### Statistical Analysis

Results in the different groups of mice were compared by ANOVA using the non-parametric Kruskal-Wallis test followed by post-testing using Dunn's multiple comparison of means. All results are presented as mean ± SEM. A statistical software package (Graph Pad Prism, San Diego, CA) was used for the analysis. P values of < 0.05 were considered statistically significant.

## Results

### Chronic OVA challenged Siglec-F deficient mice have increased levels of BAL eosinophils and peribronchial eosinophils

**Figure 1:**
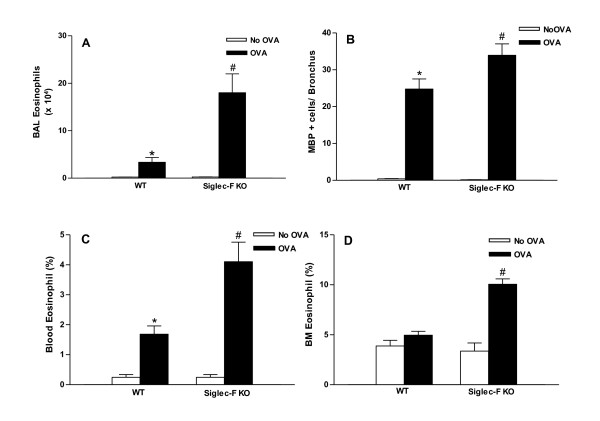
**Levels of BAL, lung, blood, and bone marrow eosinophils in Siglec-F deficient vs WT mice**. Different groups of Siglec-F deficient or WT mice were subjected to chronic OVA challenge. Non-OVA challenged mice served as a control. Eosinophils in bronchoalveolar lavage (BAL), blood, and bone marrow were quantitated in cytospin slides stained with Wright-Giemsa, whereas eosinophils in lung sections were quantitated by immunostaining with an anti-MBP Ab. Chronic OVA challenge in WT mice induced a significant increase in BAL eosinophils (p < 0.0001*)(Fig 1A), peribronchial eosinophils (p < 0.0001*)(Fig 1B), and blood eosinophils (p < 0.003*)(Fig 1C)(WT no OVA vs WT OVA). Levels of eosinophils in OVA challenged Siglec-F deficient mice were significantly increased compared to WT mice challenged with OVA in the BAL (p < 0.001^#^)(Fig 1A), lung (p < 0.04^#^)(Fig 1B), blood (p < 0.003^#^)(Fig 1C), and bone marrow (p < 0.001^#^)(Fig 1D)(n = 16 mice/group).

Chronic OVA challenge in WT mice induced a significant increase in the number of BAL eosinophils (p < 0.0001)(WT OVA vs WT no OVA)(Figure 1A), as well as a significant increase in the number of peribronchial eosinophils (p < 0.0001)(WT OVA vs WT no OVA)(Figure 1B) compared to non-OVA challenged mice. The number of BAL eosinophils in chronic OVA challenged Siglec-F deficient mice were significantly higher than that in chronic OVA challenged WT mice (18.0 ± 3.9 vs 3.4 ± 1.0 BAL eosinophils × 10^4^)(Siglec-F deficient OVA vs WT OVA)(p < 0.001)(Figure 1A). Similarly, the number of peribronchial eosinophils in chronic OVA challenged Siglec-F deficient mice were significantly higher than that in chronic OVA challenged WT mice (34.0 ± 3.1 vs 24.8 ± 2.7 MBP+ eosinophils/bronchus)(Siglec-F deficient OVA vs WT OVA)(p < 0.04)(Figure 1B). The magnitude of the increase in BAL eosinophils in Siglec-F deficient compared to WT mice (5.3 fold increase), was greater than the magnitude of the increase in peribronchial eosinophils in Siglec-F deficient compared to WT mice (1.4 fold increase).

### Chronic OVA challenged Siglec-F deficient mice have increased levels of bone marrow eosinophils and peripheral blood eosinophils

Chronic OVA challenge in WT mice induced a significant increase in the number of peripheral blood eosinophils (p < 0.003)(WT OVA vs WT no OVA)(Figure 1C), as well as a slight but statistically insignificant increase in the number of bone marrow eosinophils (p = ns)(WT OVA vs WT no OVA)(Figure 1D). The number of bone marrow eosinophils in chronic OVA challenged Siglec-F deficient mice was significantly increased compared to that of chronic OVA challenged WT mice (10.1 ± 0.5 vs 3.4 ± 0.8% eosinophils)(Siglec-F deficient OVA vs WT OVA)(p < 0.001)(Figure 1D). Similarly, the percentage of peripheral blood eosinophils in chronic OVA challenged Siglec-F deficient mice was significantly increased compared to that of chronic OVA challenged WT mice (4.1 ± 0.7 vs 1.7 ± 0.3% eosinophils)(Siglec-F deficient OVA vs WT OVA)(p < 0.003)(Figure 1C).

### Chronic OVA challenged Siglec-F deficient mice have increased levels of airway mucus

**Figure 2:**
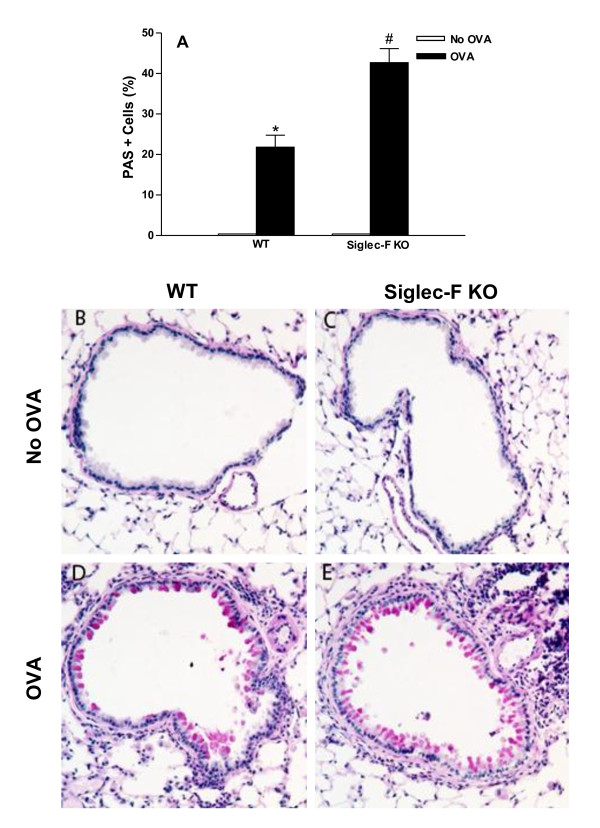
**Levels of mucus expression in Siglec-F deficient vs WT mice**. Different groups of Siglec-F deficient or WT mice were subjected to chronic OVA challenge. Non-OVA challenged mice served as a control. The level of mucus expression was quantitated in lung sections by PAS staining using a light microscope objective at 20× (Fig 2 A-E). Chronic OVA challenge in WT mice induced a significant increase in the number of PAS+ mucus cells (p = 0.0001*)(WT no OVA vs WT OVA)(Fig 2A; Fig 2B vs Fig 2D). Levels of mucus expression were significantly increased in OVA challenged Siglec-F deficient mice compared to OVA challenged WT mice (p = 0.0001^#^)(Fig 2A; Fig 2E vs Fig 2D)(n = 16 mice/group).

Chronic OVA challenge in WT mice induced a significant increase in the number of PAS+ mucus cells (p < 0.0001)(WT OVA vs WT no OVA)(Figure 2A) compared to non-OVA challenged mice. Siglec-F deficient mice challenged with OVA had a significant increase in the percentage of PAS+ mucus cells compared to OVA challenged WT mice (42.7 ± 3.5 vs 21.8 ± 3.0% PAS+ cells/bronchus)(Siglec-F deficient OVA vs WT OVA)(p < 0.0001)(Figure 2A-2E).

### Chronic OVA challenged Siglec-F deficient mice have increased levels of peribronchial fibrosis

**Figure 3:**
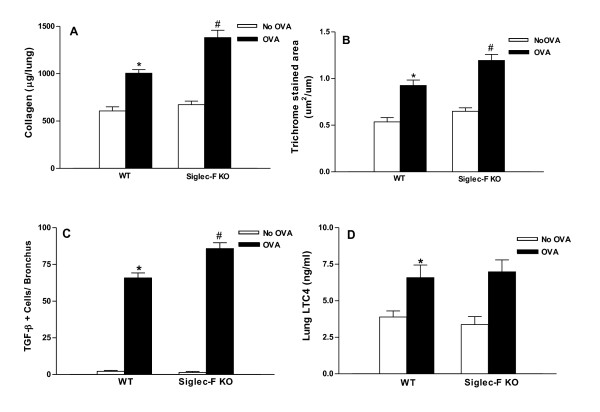
**Levels of peribronchial fibrosis, TGF-β1+ cells, and LTC4 levels in Siglec-F deficient vs WT mice**. Different groups of Siglec-F deficient or WT mice were subjected to chronic OVA challenge. Non-OVA challenged mice served as a control. Levels of peribronchial fibrosis were quantitated by assaying collagen levels in lungs (Fig 3A), as well as by quantitating the area of peribronchial trichrome staining by image analysis (Fig 3B). Levels of mediators of lung fibrosis were assessed by quantitating the number of peribronchial cells immunostaining positive for TGF-β1 (Fig 3C) as well as levels of LTC4 in BAL (Fig 3D) by Elisa. Chronic OVA challenge in WT mice induced a significant increase in lung collagen (p < 0.0001*)(Fig 3A), and the area of peribronchial trichrome staining (p < 0.0001*)(Fig 3B), the number of peribronchial TGF-β1+ cells (p < 0.0001*)(Fig 3C), and levels of BAL LTC4 (p < 0.02*)(WT no OVA vs WT OVA). Levels of lung collagen were significantly increased in OVA challenged Siglec-F deficient mice compared to WT mice challenged with OVA (p < 0.002^#^)(WT OVA vs Siglec-F OVA)(Fig 3A), as was the area of peribronchial trichrome staining (p < 0.01^#^)(Fig 3B), and the number of peribronchial TGF-β1+ cells (p < 0.001)(Fig 3C)(n = 16 mice/group).

Chronic OVA challenge in WT mice induced a significant increase in levels of peribronchial fibrosis as assessed by either increases in lung collagen (p < 0.0001)(WT OVA vs WT no OVA)(Figure 3A), or the area of peribronchial trichrome staining (p < 0.0001)(WT OVA vs WT no OVA)(Figure 3B) compared to non-OVA challenged mice. The amount of lung collagen in chronic OVA challenged Siglec-F deficient mice was significantly higher than that in chronic OVA challenged WT mice (1,382 ± 77 vs 1,005 ± 38 µg collagen/lung)(Siglec-F deficient OVA vs WT OVA)(p < 0.002)(Figure 3A).The area of peribronchial trichrome staining was also significantly higher in chronic OVA challenged Siglec-F deficient mice compared to chronic OVA challenged WT mice (p < 0.01)(Figure 3B).

### Chronic OVA challenged Siglec-F deficient mice have increased numbers of peribronchial TGF-β1+ cells but no increase in LTC4

Chronic OVA challenge in WT mice induced a significant increase in the number of peribronchial cells immunostaining positive for TGF-β1 compared to non-OVA challenged WT mice (2.2 ± 0.7 vs 65.8 ± 3.4)(WT no OVA vs WT OVA)((p < 0.0001)(Figure 3C). The number of peribronchial cells immunostaining positive for TGF-β1 in chronic OVA challenged Siglec-F deficient mice was significantly higher than that in chronic OVA challenged WT mice (p < 0.001)(Figure 3C).

Chronic OVA challenge in WT mice induced a significant increase in levels of lung LTC4 compared to non-OVA challenged mice (3.8 ± 0.4 vs 6.6 ± 0.9 ng/ml LTC4)(WT no OVA vs WT OVA)((p < 0.02)(Figure 3D). Levels of lung LTC4 in chronic OVA challenged Siglec-F deficient mice were significantly higher than in non-OVA challenged Siglec-F deficient mice (p < 0.02), but these levels were not significantly different from that noted in chronic OVA challenged WT mice (Figure 3D).

### Chronic OVA challenged Siglec-F deficient mice express increased levels of the extracellular matrix protein fibronectin

**Figure 4:**
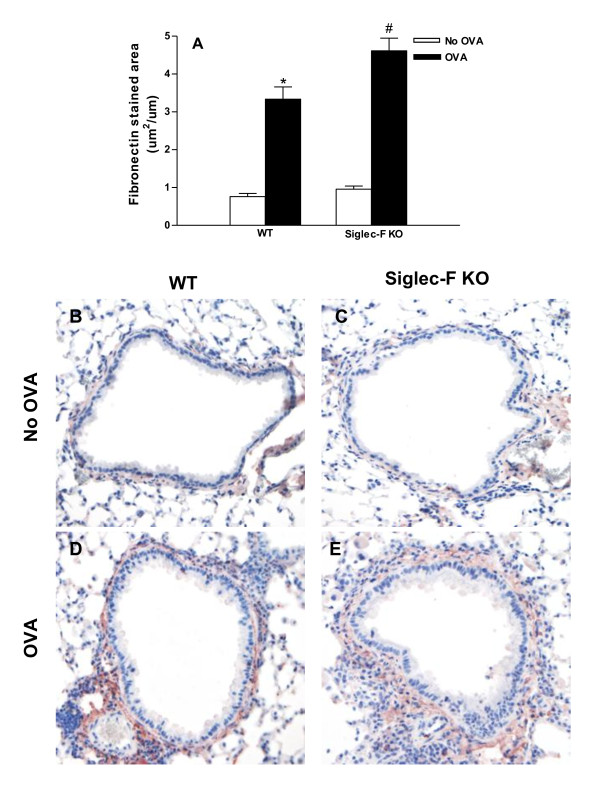
**Levels of peribronchial fibronectin in Siglec-F deficient vs WT mice**. Different groups of Siglec-F deficient or WT mice were subjected to chronic OVA challenge. Non-OVA challenged mice served as a control. Lung sections were immunostained with an anti-fibronectin Ab and the area of peribronchial fibronectin immunostaining determined by image analysis using a light microscope objective at 20× (Figure 4 A-E). Chronic OVA challenge in WT mice induced a significant increase the area of peribronchial fibronectin immunostaining (p < 0.0001*)(Fig 4A; Fig 4B vs Fig 4D)(WT no OVA vs WT OVA). The area of peribronchial fibronectin immunostaining was also significantly increased in OVA challenged Siglec-F deficient compared to OVA challenged WT mice (p < 0.01^#^)(Fig 4A; Fig 4E vs Fig 4D)(n = 16 mice/group).

Chronic OVA challenge in WT mice induced a significant increase in the area of peribronchial immunostaining of the extracellular matrix protein fibronectin compared to non-OVA challenged mice (p < 0.0001)(Figure 4A). The area of peribronchial fibronectin immunostaining in chronic OVA challenged Siglec-F deficient mice was significantly higher than that in chronic OVA challenged WT mice (4.6 ± 0.3 vs 3.3 ± 0.3)(Siglec-F deficient OVA vs WT OVA)(p < 0.01)(Figure 4A-4E.

### Levels of lung cytokines and chemokines in chronic OVA challenged Siglec-F deficient mice

**Figure 5:**
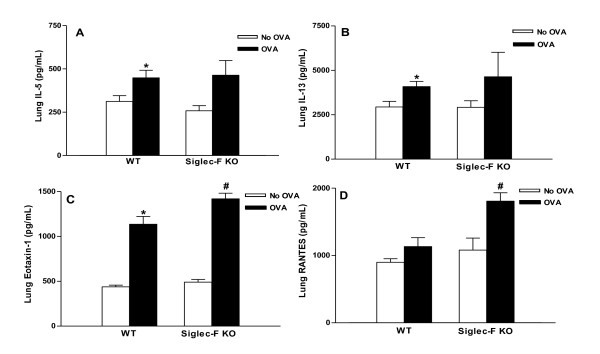
**Levels of lung cytokines and chemokines in Siglec-F deficient vs WT mice**. Different groups of Siglec-F deficient or WT mice were subjected to chronic OVA challenge. Non-OVA challenged mice served as a control. Levels of IL-5 (Fig 5A), IL-13 (Fig 5B), eotaxin-1 (Fig 5C), and RANTES (Fig 5D) were measured in lung by ELISA. Chronic OVA challenge in WT mice induced a significant increase in IL-5 (p < 0.03*)(Fig 5A), IL-13 (p < 0.04*)(Fig 5B), and eotaxin-1 (p < 0.01*)(Fig 5C), but not RANTES (p = ns)(Fig 5D). Levels of IL-5 (Fig 5A) and IL-13 (Fig 5B) were no different in OVA challenged Siglec-F deficient mice compared to WT mice challenged with OVA, while levels of eotaxin-1 (p < 0.04^#^) (Fig 5C) and RANTES (p < 0.02^#^)(Fig 5D) were increased in Siglec-F deficient mice compared to WT mice challenged with OVA (n = 16 mice/group).

Chronic OVA challenge in WT mice induced a significant increase in lung levels of IL-5 (p < 0.03)(Figure 5A), IL-13 (p < 0.04)(Figure 5B), and eotaxin-1 (p < 0.01)(Figure 5C) compared to WT mice not challenged with OVA. The increase in levels of lung RANTES in WT mice following OVA challenge was not statistically significant (899 ± 53 vs 1,132 ± 134)(WT no OVA vs WT OVA)(p = ns).

Levels of lung IL-5 (Figure 5A) and IL-13 (Figure 5B) were similar in OVA challenged Siglec-F deficient and OVA challenged WT mice. Levels of lung eotaxin-1 (p < 0.04)(Figure 5C), and RANTES (p < 0.02)(Figure 5D) were increased in Siglec-F deficient compared to WT mice challenged with OVA.

### Chronic OVA challenged Siglec-F deficient mice express increased levels of peribronchial Siglec-F ligands

**Figure 6:**
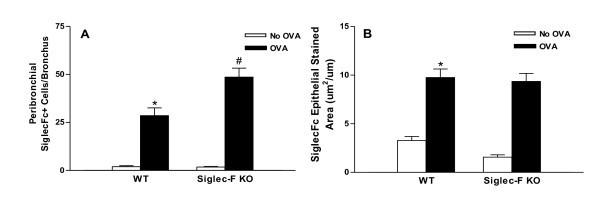
**Quantitation of Siglec-F ligands in airway epithelial cells and peribronchial inflammatory cells**. Lung sections from non-OVA or chronic OVA challenged WT or Siglec-F deficient mice were immunostained with a Siglec-F-Fc or a control Fc. The number of peribronchial cells (Fig 6A), as well as the area of airway epithelial cells (Fig 6B) immunostaining positive for Siglec-F-Fc was quantitated by image analysis. OVA challenged WT mice had significantly increased numbers of peribronchial cells immunostaining positive with the Siglec-F-Fc (p = 0.0001*)(Fig 6A) and significantly increased levels of airway epithelial cell Siglec-F-Fc immunostaining compared to non-OVA challenged WT mice (p = 0.0001*)(Fig 6B). Siglec-F deficient mice challenged with OVA had significantly increased numbers of peribronchial cells immunostaining positive for Siglec-F-Fc compared to OVA challenged WT mice (p = 0.01^#^)(Fig 6A), whereas airway epithelial Siglec-F-Fc immunostaining was similar in OVA challenged Siglec-F deficient and WT mice (Fig 6B)(n = 16 mice/group).

Image analysis quantitation demonstrated that there was a significant increase in the number of peribronchial cells immunostaining positive with the Siglec-F-Fc in WT mice exposed to chronic OVA challenge compared to non-OVA challenged WT mice (p < 0.0001)(Figure 6A). Similarly, the area of airway epithelium immunostaining positive with the Siglec-F-Fc demonstrated a significant increase in WT mice exposed to chronic OVA challenge compared to non-OVA challenged WT mice (p < 0.0001)(Figure 6B). Lung sections immunostained with the control R114A Siglec-F-Fc did not detect positive staining of peribronchial cells or airway epithelial cells.

The number of peribronchial cells immunostaining positive with the Siglec-F-Fc was significantly higher in chronic OVA challenged Siglec-F deficient mice compared to chronic OVA challenged WT mice (48.6 ± 4.7 vs 28.5 ± 4.1 peribronchial Siglec-Fc+ cells) (Siglec-F deficient OVA vs WT OVA)(p < 0.01)(Figure 6A). In contrast, the area of airway epithelial immunostaining positive with the Siglec-F-Fc in chronic OVA challenged Siglec-F deficient mice was similar to that observed in chronic OVA challenged WT mice (Siglec-F deficient OVA vs WT OVA)(p = ns)(Figure 6B).

### IL-4 and IL-13 up-regulate expression of Siglec-F ligands in WT and Siglec-F deficient mice

**Figure 7:**
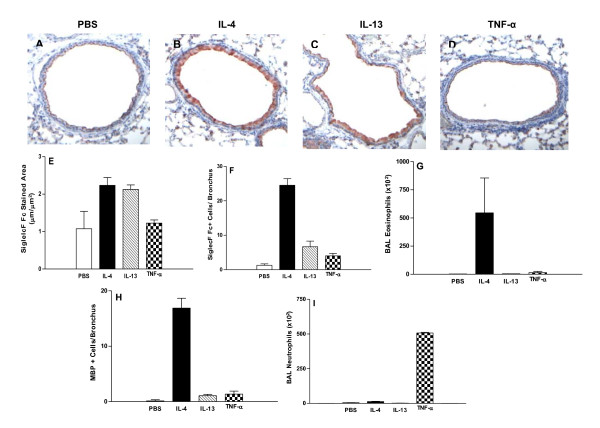
**Quantitation of Siglec-F ligands in airway epithelial cells and peribronchial inflammatory cells in WT and Siglec-F deficient mice challenged with IL-4, IL-13 or TNF-α**. WT or Siglec-F deficient mice were administered either IL-4, IL-13, TNF-α, or PBS diluent control. Twenty four hours after each individual cytokine or diluent challenge, the mice were sacrificed. BAL was obtained for determination of eosinophil and neutrophil cell counts, and the lungs were processed for immunohistology to detect Siglec-F ligand expression and MBP+ peribronchial eosinophils using a light microscope objective at 20×. Administration of either IL-4 (p < 0.001, WT; p < 0.001, Siglec-F deficient) or IL-13 (p < 0.001, WT; p < 0.001, Siglec-F deficient) induced a similar significant increase in levels of Siglec-F ligand expression by peribronchial cells (Fig 7A-C, 7E). In contrast, administration of TNF-α induced a small increase in peribronchial Siglec-F ligands (p < 0.01)(Fig 7E), but did not significantly increase Siglec-F ligand expression by airway epithelium in either WT or Siglec-F deficient mice (p = ns) (Fig 7 D, 7F). Administration of either IL-4 (p < 0.001 WT; p < 0.001 Siglec-F deficient) or IL-13 (p < 0.001 WT; p < 0.001 Siglec-F deficient) induced significantly increased levels of Siglec-F ligand expression by airway epithelium (Fig 7A-C, 7F). Although both IL-4 and IL-13 induced strong upregulation of Siglec-F ligand expression by airway epithelium (Fig 7F), levels of Siglec-F ligands in airway epithelium were slightly lower in Siglec-F deficient vs WT mice induced by IL-13 (p = 0.02) but not IL-4 (p = 0.10) (Fig 7F). IL-4 induced the strongest eosinophil response in BAL (WT p < 0.03; Siglec-F deficient p = 0.02)(Fig 7G) and lung (WT p < 0.001; Siglec-F deficient p < 0.001)(Fig 7H), while IL-13 and TNF-α induced a weaker eosinophil response in BAL (Fig 7G) and lung (Fig 7H). TNF-α, but not IL-4 or IL-13, induced a strong neutrophil response in BAL in both WT (p = 0.03) and Siglec-F deficient mice (p = 0.03) (Fig 7I)(n = 3 mice/group).

In WT mice administration of either IL-4 (p < 0.001) or IL-13 (p < 0.001) induced significantly increased levels of Siglec-F ligand expression by peribronchial cells and airway epithelium compared to control diluent challenge (Figure 7A-C, 7E-F). In contrast, administration of TNF-α (p = ns) did not induce significant Siglec-F ligand expression by airway epithelium (p = ns) but induced a slight but statistically significant increase in Siglec-F ligand expressing peribronchial cells (p < 0.01) (Fig. 7E-F).

In Siglec-F deficient mice, similar results were observed with both IL-4 and IL-13 inducing increased Siglec-F ligand expression to a similar degree in both peribronchial cells (Figure 7E) and airway epithelium (Figure 7F), while TNFα did not significantly induce Siglec-F ligand expression in airway epithelium (Figure 7E and 7F). In comparing WT and Siglec-F deficient mice, responses to these three cytokines were similar in the number of Siglec-F ligand+ peribronchial cells (Figure 7E). Although both IL-4 and IL-13 induced strong upregulation of Siglec-F ligand expression by airway epithelium in both WT and Siglec-F deficient mice (Figure 7F), levels of Siglec-F ligands in airway epithelium were slightly lower in Siglec-F deficient vs WT mice induced by IL-13 (p = 0.02) but not IL-4 (p = 0.10) (Figure 7F). The number of MBP+ peribronchial cells were higher in Siglec-F deficient mice compared to WT mice administered IL-4 (p = 0.05) or IL-13 (p = 0.01)(Figure 7H). Administration of TNFα induced a significant BAL neutrophil response in both WT (p = 0.03)(Figure 7I) and Siglec-F deficient mice (p = 0.03)(Figure 7I). In contrast, IL-4 and IL-13 induced a strong BAL eosinophil response in both WT and Siglec-F deficient mice (7G).

### Chronic OVA challenged Siglec-F deficient mice have increased smooth muscle thickness

**Figure 8:**
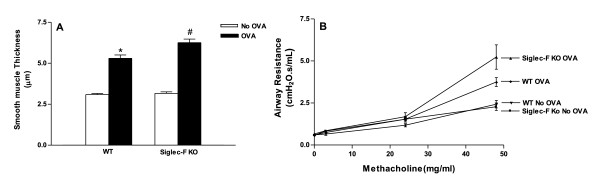
**Thickness of the peribronchial smooth muscle layer and airway responsiveness in Siglec-F deficient vs WT mice**. Different groups of Siglec-F deficient or WT mice were subjected to chronic OVA challenge. Non-OVA challenged mice served as a control. The thickness of the peribronchial smooth muscle layer was quantitated in lung sections (Fig 8A). Chronic OVA challenge in WT mice induced a significant increase in the thickness of the peribronchial smooth muscle layer (p = 0.0001*)(Fig 8A)(WT no OVA vs WT OVA). The thickness of the peribronchial smooth muscle layer in OVA challenged Siglec-F deficient mice was significantly increased compared to WT mice challenged with OVA (p = 0.003^#^)(Fig 6A). Airway resistance (Raw) was measured (cm H_2_O.s/ml) in different groups of intubated and ventilated Siglec-F deficient deficient or WT mice following nebulization of either PBS diluent or MCh (3, 24, 48 mg/ml)(Fig 8B). Chronic OVA challenge in WT mice induced a significant increase in airway resistance (WT no OVA vs WT OVA; p < 0.002, 48 mg/ml MCh)(Fig 6B). Siglec-F deficient mice challenged with OVA had a statistically insignificant trend for increased airway responsiveness compared to WT mice (Siglec-F deficient OVA vs WT OVA; p = 0.15, 48 mg/ml MCh)(n = 16 mice/group).

Chronic OVA challenge in WT mice induced a significant increase in the thickness of the peribronchial smooth muscle layer (5.3 ± 0.2 vs 3.1 ± 0.1 µm)(p < 0.0001)(WT OVA vs WT no OVA)(Figure 8A) compared to non-OVA challenged WT mice. Siglec-F deficient mice challenged chronically with OVA had a modest (approximately 20%), but statistically significant, increase in the thickness of the peribronchial smooth muscle layer (6.3 ± 0.2 vs 5.3 ± 0.2 µm)(p < 0.003) (Siglec-F deficient mice OVA vs WT OVA)(Figure 8A) compared to OVA challenged WT mice.

### Chronic OVA challenged Siglec-F deficient mice and AHR

Chronic OVA challenge in WT mice induced a statistically significant increase in airway responsiveness to Mch (WT OVA vs WT no OVA, Mch 48 mg/ml)(p < 0.002)(Figure 8B). Siglec-F deficient mice challenged with OVA had a small but statistically insignificant increase in airway responsiveness compared to WT mice challenged with OVA (Siglec-F deficient mice OVA vs WT mice OVA; Mch 48 mg/ml)(p = 0.15)(Figure 8B).

## Discussion

In this study we have utilized Siglec-F deficient mice to demonstrate an important role for Siglec-F in modulating levels of airway remodeling (peribronchial fibrosis, thickness of the smooth muscle layer), mucus expression, deposition of extracellular matrix proteins such as fibronectin, as well as an important role for Siglec-F in regulating levels of chronic allergen induced peribronchial Siglec-F ligands. This study confirms the importance of Siglec-F in airway remodeling and extends results obtained using an anti-Siglec-F Ab in WT mice [15], to demonstrate an important role for Siglec-F in mucus expression, deposition of extracellular matrix proteins such as fibronectin, and regulating levels of chronic allergen induced peribronchial Siglec-F ligands which were not demonstrated in previous studies using an anti-Siglec-F Ab in WT mice [15]. In addition, we demonstrated that Th2 cytokines such as IL-4 or IL-13 induce equivalent upregulation of Siglec-F ligand expression by airway epithelium in vivo. In contrast, TNF-α another cytokine expressed in the remodeled airway does not significantly regulate airway epithelial Siglec-F ligand expression, even though it induced a significant BAL neutrophil response. In addition to airway epithelium we have previously demonstrated that eosinophils also express Siglec-F ligands [5]. All three cytokines (IL-4, IL-13, and TNF-α) increased the number of peribronchial cells expressing Siglec-F ligands and this was proportional to the induced lung eosinophil response which was strongest with IL-4 and IL-13 and significantly weaker with TNF-α. As activating Siglec-F receptors with anti-Siglec-F antibodies in vitro induces apoptosis [5,14-16], the up-regulated expression of Siglec-F ligands by airway epithelium in response to Th2 cytokines may be a mechanism by which airway epithelium downregulates eosinophilic inflammation when eosinophils come in contact with the airway epithelium. In vitro studies have demonstrated that a synthetic Siglec-8 ligand induces human eosinophil apoptosis [24] underscoring the potential therapeutic utility of using Siglec ligands to limit eosinophilic inflammation. Chronic OVA challenge induced a similar significant increase in levels of lung IL-5 and IL-13 in Siglec-F deficient and WT mice. However there was a slight but statistically significant increase in levels of lung eotaxin-1 and RANTES in chronic OVA challenged Siglec-F deficient compared to WT mice which may reflect the increased numbers of peribronchial inflammatory cells in chronic OVA challenged Siglec-F deficient capable of expressing these chemokines. This study also demonstrated that the important eosinophil product LTC4, a mediator not investigated in previous studies using an anti-Siglec-F Ab in WT mice [15], was unlikely to be contributing to enhanced remodeling in Siglec-F deficient mice. Siglec-F deficient mice challenged with chronic allergen had significantly enhanced peribronchial fibrosis, as well as increased mucus expression, and an increase in the thickness of the smooth muscle layer. Although Siglec-F deficient mice had increased thickness of the smooth muscle layer, the trend for increased airway responsiveness was not statistically significant.

The mechanism by which Siglec-F deficient mice have enhanced airway remodeling is not due to a direct effect of Siglec-F on fibroblasts, epithelial cells, or smooth muscle as these cells do not express Siglec-F. In the absence of an allergen stimulus to induce airway inflammation, non-OVA challenged Siglec-F deficient mice do not have evidence of airway remodeling, again supporting the concept that it is the exaggerated inflammatory response in Siglec-F deficient mice, rather than structural cells in the airway, that are the primary initiator of enhanced airway remodeling in Siglec-F deficient mice. Siglec-F is most highly expressed on eosinophils [5], but can also be detected on macrophages and activated CD4+ T cells as previously demonstrated [5]. As Siglec-F deficiency results in increased numbers of eosinophils in the allergen challenged lung, one potential explanation for the enhanced airway remodeling in Siglec-F deficient mice is the increased numbers of eosinophils in the lung expressing pro-fibrotic growth factors that may contribute to remodeling including TGF-β1 [7,22]. In support of this hypothesis are the increased numbers of TGF-β1+ cells we have identified in Siglec-F deficient mice. The importance of eosinophils and TGF-β1 to airway remodeling is suggested from murine studies in which airway remodeling is significantly reduced in mice treated with an anti-TGF-β1 Ab [25], as well as in Smad 3 deficient mice [22] which have impaired TGF-β signaling. In addition studies in IL-5 deficient mice [7] as well as in human subjects with asthma treated with anti-IL-5 [17] demonstrate reduced numbers of eosinophils, reduced TGF-β1+ eosinophils, and reduced airway remodeling. Although several studies show an important role for eosinophils, TGF-β1, and Smad signaling in airway remodeling [22,25], there are studies which have demonstrated that an anti-TGF-β1 Ab does not reduce remodeling in mice [26], and that an anti-TGF-β1 Ab increase airway hyperreactivity in mice [27]. Increased numbers of peribronchial cells expressing TGF-β1 in chronic OVA challenged Siglec-F decient mice may also contribute to increased smooth muscle thickness as TGF-β1 induces airway smooth muscle hypertrophy [28]. As LTC4 is expressed by eosinophils [19] and has pro-remodeling potential in asthma [20], we examined whether Siglec-F deficient mice had increased levels of lung LTC4 in their remodeled airways. As there was no significant difference in levels of LTC4 in the remodeled airways of WT and Siglec-F deficient mice, LTC4 is unlikely to explain the differences in levels of airway remodeling in WT and Siglec-F deficient mice. The mechanism of increased mucus expression in chronic OVA challenged Siglec-F decient mice at present unknown. One possible explanation is that the increased numbers of eosinophils in the airway in Siglec-F deficient mice express increased levels of mediators that can influence mucus secretion including granule mediators (ECP, MBP)[29,30], lipid mediators (LTC4)[31], cytokines (IL-13)[31], or other at present unknown mediators. Alternatively eosinophils may release a mediator that influences a second cell type to subsequently influence mucus expression. Our initial experiments to address this question demonstrate no differences in levels of IL-13 or LTC4 between Siglec-F deficient and WT mice.

In this study we also made the novel observation that Siglec-F deficient mice had increased levels of peribronchial extracellular matrix remodeling as indicated by increased fibronectin deposition. Fibronectin is a large extracellular matrix glycoprotein molecule consisting of two similar subunits of 220-250 kDa [32] that has been detected in increased amounts in the remodeled airway in human asthma [33], as well as in fatal asthma [34]. There are two forms of fibronectin, plasma fibronectin (dimeric and soluble) and cellular or extracellular matrix fibronectin (multimeric and insoluble)[32]. Extracellular matrix deposition of fibronectin may enhance airway remodeling in asthma by contributing to the formation of collagen fibrils [35], mediate the migration of fibroblasts [36], and increase the proliferation of smooth muscle cells [37]. In addition, fibronectin may enhance eosinophilc inflammation in the airway through its ability to increase CC chemokine expression by airway smooth muscle cells [38].

In this study we demonstrated that Siglec-F plays an important role in mediating several key features of airway remodeling but did not a play a significant role in mediating AHR. Although mathematical models of asthma predict that the increased airway wall thickening in remodeled airways would result in disproportionately severe airway narrowing and responsiveness [39], studies in human asthmatics have demonstrated that airway wall remodeling and thickening in asthma is associated with reduced rather than increased airway reactivity to MCh [40]. One potential explanation suggested [41] for the discrepancy in results between the mathematical modeling studies and the computerized tomography scan studies in asthma is that the mathematical modeling studies did not fully take into account the potential effect of airway wall thickening on the mechanical properties of the airway, e.g., stiffness of the airway [41]. Our studies with Siglec-F-deficient mice underscore the fact that a gene such as Siglec-F, which plays a significant role in mediating several important aspects of airway remodeling, may not play an essential role in mediating AHR. Our studies using an anti-Siglec-F Ab in a model of chronic OVA allergen induced airway remodeling also demonstrated inhibition of airway remodeling but no effect on AHR measurements [15], consistent with our observations in this study using Siglec-F deficient mice.

In addition to investigating the effect of Siglec-F on airway remodeling we examined whether the presence versus absence of Siglec-F receptors influenced levels of Siglec-F ligands being expressed. We have previously demonstrated that levels of Siglec-F are upregulated on blood eosinophils following OVA challenge [5]. In this study we examined whether removal of the Siglec-F receptor in Siglec-F deficient mice influenced levels of Siglec-F ligand expression. Our study demonstrates that the number of Siglec-F ligand+ peribronchial cells was significantly increased in OVA challenged Siglec-F deficient compared to OVA challenged WT mice. This increase in level of peribronchial Siglec-F ligands in Siglec-F deficient mice is most likely accounted for by the increased numbers of inflammatory cells expressing Siglec-F ligands (i.e. eosinophils, macrophages) recruited to the airway following OVA challenge, though we cannot rule out a contribution from upregulation of Siglec-F ligand expression by inflammatory cells recruited to the lung. In contrast, constitutive and OVA induced levels of expression of the Siglec-F ligand in airway epithelium are similar in Siglec-F deficient and WT mice.

## Conclusions

Overall, this study demonstrates an important role for Siglec-F in modulating levels of chronic eosinophilic airway inflammation, peribronchial fibrosis, thickness of the smooth muscle layer, fibronectin deposition, mucus expression, and levels of peribronchial Siglec-F ligands suggesting that Siglec-F may normally function to limit levels of chronic eosinophilic inflammation, mucus, and remodeling. As Siglec-F is not expressed on structural cells in the lung such as fibroblasts, epithelial cells, or smooth muscle cells, the enhanced remodeling is likely due to enhanced airway inflammation in Siglec-F deficient mice. Siglec-F deficient mice express significantly increased numbers of peribronchial TGF-β1+ cell (but not increased amounts of LTC4) suggesting that TGF-β1 may be one possible mechanism of enhanced airway remodeling in these mutant mice. In WT and Siglec-F deficient mice airway epithelial Siglec-F ligand expression can be up-regulated by Th2 cytokines such as IL-4 or IL-13, but not by TNF-α, suggesting a potential mechanism for airway epithelium to down regulate eosinophilic inflammation. Insights from these studies of Siglec-F deficient mice may have application to understanding the role of the human paralog of Siglec-F (i.e. Siglec-8) [42] in humans with asthma and airway remodeling.

## List of abbreviations

ECP: Eosinophil Cationic Protein; LTC4: Leukotriene C4: MBP: Major basic protein; OVA: Ovalbumin; PAS: Periodic acid Schiff; Siglec-F: **Si**alic acid-binding **Ig**-superfamily **lec**tin-F; TGF-β1: Transforming growth factor beta 1.

## Competing interests

The authors declare that they have no competing interests.

## Authors' contributions

JYC, DJS, AP, PS, TD, and MM made significant contributions to design, acquisition of data, as well as analysis and interpretation of data. SD made significant contribution to acquisition of data. AV and DHB made significant contributions to conception, design, analysis and interpretation of data, and drafting of manuscript.
